# Retrospective Analysis of Historical *Listeria monocytogenes* Clinical Isolates, New York, USA, 2000–2021^1^

**DOI:** 10.3201/eid3110.241876

**Published:** 2025-10

**Authors:** Hilal Samut, Damaris V. Mendez-Vallellanes, Hannah Hoyt, Samantha E. Wirth, Lisa Mingle, Brian D. Sauders, Gregory A. Deiulio, Alyssa W. Dickey, Maria L. Ishida, William J. Wolfgang, Martin Wiedmann, Renato H. Orsi

**Affiliations:** Cornell University, Ithaca, New York, USA (H. Samut, M. Wiedmann, R.H. Orsi); Wadsworth Center, New York State Department of Health, Albany, New York, USA (D. V. Mendez-Vallellanes, H. Hoyt, S.E. Wirth, L. Mingle, W.J. Wolfgang); New York State Department of Agriculture and Markets, Albany (B.D. Sauders, G.A. Deiulio, A.W. Dickey, M.L. Ishida)

**Keywords:** Listeria monocytogenes, listeriosis, bacteria, food safety, enteric infections, cluster, foodborne, surveillance, epidemiology, persistence, outbreak, REP strains, cgMLST, New York, United States

## Abstract

We used whole-genome sequencing data to investigate historical *Listeria monocytogenes* clinical (n = 1,046) and nonclinical (n = 1,325) isolates from New York, USA. Applying a threshold of <20 single-nucleotide polymorphism differences for single-linkage clustering, 321 clinical isolates clustered into 85 clusters ranging from 2–33 isolates per cluster. Fourteen clusters included isolates with outbreak codes (4 clusters with New York codes and 10 with multistate codes). Three New York outbreak codes were assigned to isolates that were genetically highly related and from cases <2 months apart. Fifteen clusters included isolates that were obtained >10 years apart; 33 clusters included isolates from the same or contiguous counties. Seventeen clusters included food and environmental isolates highly related to clinical isolates. These findings suggest that some listeriosis clusters can be local and span a long period, demonstrating the importance of investigating small, localized listeriosis cases with closely related isolates, even over long timeframes.

*Listeria monocytogenes* contamination affects various food commodities, including dairy products (e.g., cheeses, ice cream), ready-to-eat meats (e.g., deli meats), fruits, and vegetables. The ability to survive under adverse conditions enables *L. monocytogenes* to persist in food processing environments over extended periods (*1*), which can lead to recurrent contamination of finished, ready-to-eat food products.

*L. monocytogenes* outbreak investigations pose challenges because of long incubation time, small outbreak size, and long timespan between cases (*2*). Identifying the source becomes even more difficult when genetically related environmental and food isolates are geographically widespread or associated with multiple food commodities or environmental sources (*3*). Those challenges complicate understanding of *L. monocytogenes* transmission patterns, often resulting in the detection of primarily large or localized outbreaks (*2*) and the characterization of most listeriosis cases as sporadic.

With the advent of high-throughput DNA sequencing, the Centers for Disease Control and Prevention (CDC) PulseNet and the US Food and Drug Administration GenomeTrackr programs started the routine use of whole-genome sequencing (WGS) as a subtyping tool in *L. monocytogenes* outbreak surveillance in September 2013. This tool has enabled public health agencies to connect cases over extended periods, refine case definitions, connect sporadic cases to sources, and link cases to common sources using WGS data from food and environmental isolates obtained through surveillance efforts (*2*,*4*). In this study, we sequenced historical *L. monocytogenes* clinical isolates obtained in New York, USA, during 2000–2021 to retrospectively analyze listeriosis cases with the aim of assessing temporal and geographic distribution of clustered isolates and patterns associated with clusters investigated by the New York State Department of Health (NYSDOH) and CDC.

## Materials and Methods

We have provided a complete description of methods in the Appendix. In brief, we performed WGS on 957 *L. monocytogenes* clinical isolates collected by NYSDOH (2000–2021) and 89 by the New York City Department of Health and Mental Hygiene (2000–2004) using the Illumina NextSeq 500 platform (Illumina, https://www.illumina.com). We sequenced 802 other nonclinical isolates (i.e., food and environmental) (2000–2021) using Illumina MiSeq and obtained WGS data for 523 nonclinical isolates (2000–2020) from previous studies (*5*,*6*). We submitted all data to PulseNet and the National Center for Biotechnology Information Pathogen Detection database, where single-nucleotide polymorphism (SNP) clusters and pairwise SNP distances were generated (*7*).

We obtained metadata (e.g., collection date, county, source) from Food Microbe Tracker, NYSDOH, New York City Department of Health and Mental Hygiene, and New York State Department of Agriculture and Markets. We filtered duplicate sequences of the same isolate and excluded 40 clinical isolates from mother–child pairs or the same patient. Clinical isolates were clustered into New York clusters using single-linkage at <20 SNPs (*8*–*10*); nonclinical isolates within <50 SNPs of a cluster were added (*11*). We classified clusters by county proximity (i.e., same, contiguous, or noncontiguous).

We performed core-genome multilocus sequence typing (cgMLST) in PulseNet 2.0 with automated quality control, SPAdes assemblies (https://ablab.github.io/spades), and allele calling against 1,748 loci. We performed statistical analyses in R version 4.2.1 (The R Project for Statistical Computing, https://www.r-project.org) using generalized least square to assess pairwise SNP distances within clusters and generalized linear model with negative binomial distribution for predicting maximum SNP distances between clusters. We reported the estimated mean differences in pairwise SNP distances (β_pairwise_) and maximum SNP distance between clusters (β_cluster_).

## Results

### Cluster Size

**Figure 1:**
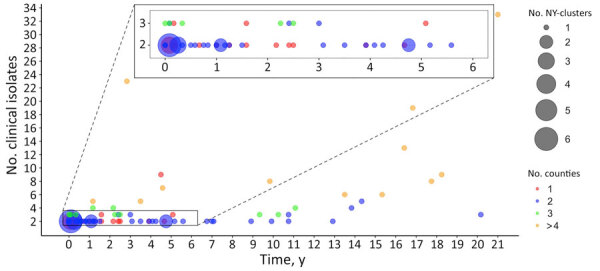
Distribution of timespan, number of clinical isolates, and number of counties among clusters in retrospective analysis of historical *Listeria monocytogenes* clinical isolates, New York, USA, 2000–2021. Clinical isolates that show a single-nucleotide polymorphism (SNP) distance of <20 SNPs to >1 of the other clinical isolates were grouped into a cluster; therefore, each cluster contains >2 clinical isolates. Each circle represents between 1 and 6 clusters, and the size of the circle is proportional to the number of clusters represented. Circles are color-coded according to the number of counties in each cluster. Timespan represents the interval between the first and the last collected clinical isolates in a cluster, measured in years with a month (1/12 of a year) as the minimum measurable unit. If the first and last collected clinical isolates in a cluster were obtained within the same month of the same year, the timespan for the cluster is zero.

In total, 1,046 clinical isolates were obtained in New York during 2000–2021. Although 725 (69%) of the isolates showed >20 SNP differences from another New York clinical isolate, 321 (31%) showed <20 SNP differences from >1 other New York clinical isolate; those closely-related isolates were grouped into clusters such that a cluster included >2 clinical isolates differing by <20 SNPs, for a total of 85 clusters (median of 2 isolates per cluster of 2–isolates) (Figure 1).

We divided clusters into 3 arbitrary categories: small (2 or 3 clinical isolates), medium (4–10 clinical isolates), and large (>10 clinical isolates). Of the 85 clusters, 67 (79%) clusters were small, 14 (16%) were medium, and 4 (5%) were large, suggesting that most potential outbreaks involve 2–3 identified cases only.

### Timespan of Clusters

The average timespan (i.e., time interval between the collection dates of the first and last collected clinical isolates in a cluster) among 85 clusters was 4.59 years (median 2.42 years, interquartile range [IQR] 0.33–6.75 years) (Figure 1); the range was 0 months (observed in 4 small clusters) to 21 years (covering the entire study period). Moreover, 15 clusters (18%) exhibited timespans >10 years, underscoring the diversity in temporal dynamics of the clinical isolates.

Although 39 (46%) clusters lasted relatively short durations of <2 years, only 89 (28%) of the clinical isolates were represented in those clusters. Conversely, most clusters’ clinical isolates (72%, 232) were grouped in clusters (54%, 46) that lasted >2 years. Of the 46 clusters spanning >2 years, 30 (65%) were small clusters, and 5 small clusters extended beyond 10 years (Appendix Table 1), suggesting the presence of long-lasting small outbreaks in New York during the study period.

Clusters including isolates exclusively obtained before 2014 (n = 41), when pulsed-field gel electrophoresis (PFGE) was the only subtyping method for *L. monocytogenes* clinical isolates in New York, were significantly associated with their isolates not having NYSDOH-assigned cluster codes (p<0.001 by Pearson χ^2^ test with Yates’ continuity correction) (Appendix Table 1). Moreover, although 13 of the 20 New York clusters including isolates exclusively obtained in or after 2014 (when WGS was first used alongside with PFGE as a subtyping method for *L. monocytogenes* clinical isolates in New York) had all their isolates assigned a NYSDOH cluster code, only 1 out of 65 clusters with >1 isolate obtained before 2014 had all its isolates assigned a NYSDOH cluster code.

### County Origin of Isolates

**Figure 2:**
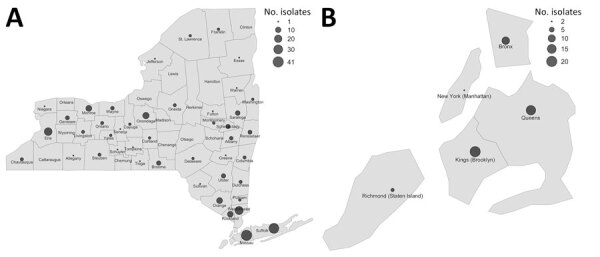
Distribution of clinical isolates within clusters (i.e., the isolates with pairwise SNP distance ≤20) in retrospective analysis of historical *Listeria monocytogenes* clinical isolates, New York, USA, 2000–2021. A) Distribution of isolates by New York county during 2000–2021 (except for the New York City [NYC] area); B) distribute of isolates in NYC during 2000–2004. The boroughs of NYC (Manhattan, Brooklyn, Queens, the Bronx, and Staten Island) were separated from New York counties outside NYC because of the limited number of isolates available for these regions.

The 1,046 clinical isolates were obtained from 59 of 62 New York counties (no isolates from Hamilton, Herkimer, and Lewis Counties); clusters included isolates from 49 counties (Figure 2, panels A, B). Excluding the 5 New York City (NYC) boroughs (Figure 2, panel A), Nassau (n = 41) had the highest number of isolates, followed by Suffolk (n = 38), Westchester (n = 24), Erie (n = 22), Onondaga (n = 14), and Monroe (n = 12) Counties (Appendix Table 2). During January 2000–September 2021, the highest incidence rates were recorded in Yates and Genesee Counties; rates were 86.9/1,000,000 persons over the study time span in Yates County and 81.8 isolates/1,000,000 persons over the study time span in Genesee County (data not shown).

Fifty-two clusters (61%) consisted of isolates collected from noncontiguous counties; 38 were small clusters (Appendix Table 1). Excluding NYC, most isolates were obtained in the Metropolitan area, which includes Long Island and the Hudson Valley. The Central region had the lowest number of *L. monocytogenes* isolates in this study (Appendix Table 3). Excluding NYC isolates, we observed no significant association between cluster size and the regions where clinical isolates were recorded (p = 0.83 by Pearson χ^2^ test) (Appendix Table 3).

A total of 33 (39%) clusters included isolates from the same or contiguous counties (Appendix Table 1). Among the 18 clusters from the same county, 17 were small clusters. One medium cluster of 9 clinical isolates (NY-cluster 18) with all isolates from the same county had a timespan of 4.5 years (Appendix Table 1), and the isolates were all highly related (SNP distances ranging from 0 to 6); although the 3 isolates collected during June–October 2014 were assigned 1 outbreak code, the other 6 isolates, collected during June 2016–December 2018, were assigned a different outbreak code (Appendix Table 4).

### SNP Differences in Clinical Isolates

**Figure 3:**
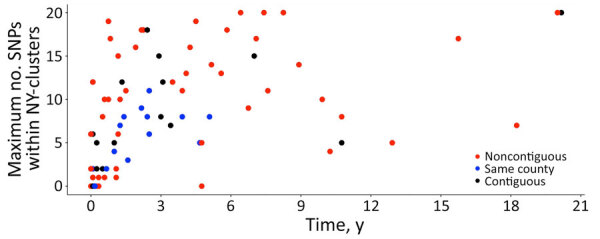
Maximum SNP distances observed in clusters in retrospective analysis of historical *Listeria monocytogenes* clinical isolates, New York, USA, 2000–2021. Timespan corresponds to the interval in years between an isolate pair where the maximum number of SNPs was observed among closely related isolates (i.e., SNPs <20). If the clinical isolates in a NY-cluster were collected within the same month of the same year, the timespan for the NY-cluster is 0. SNP, single-nucleotide polymorphism.

**Figure 4:**
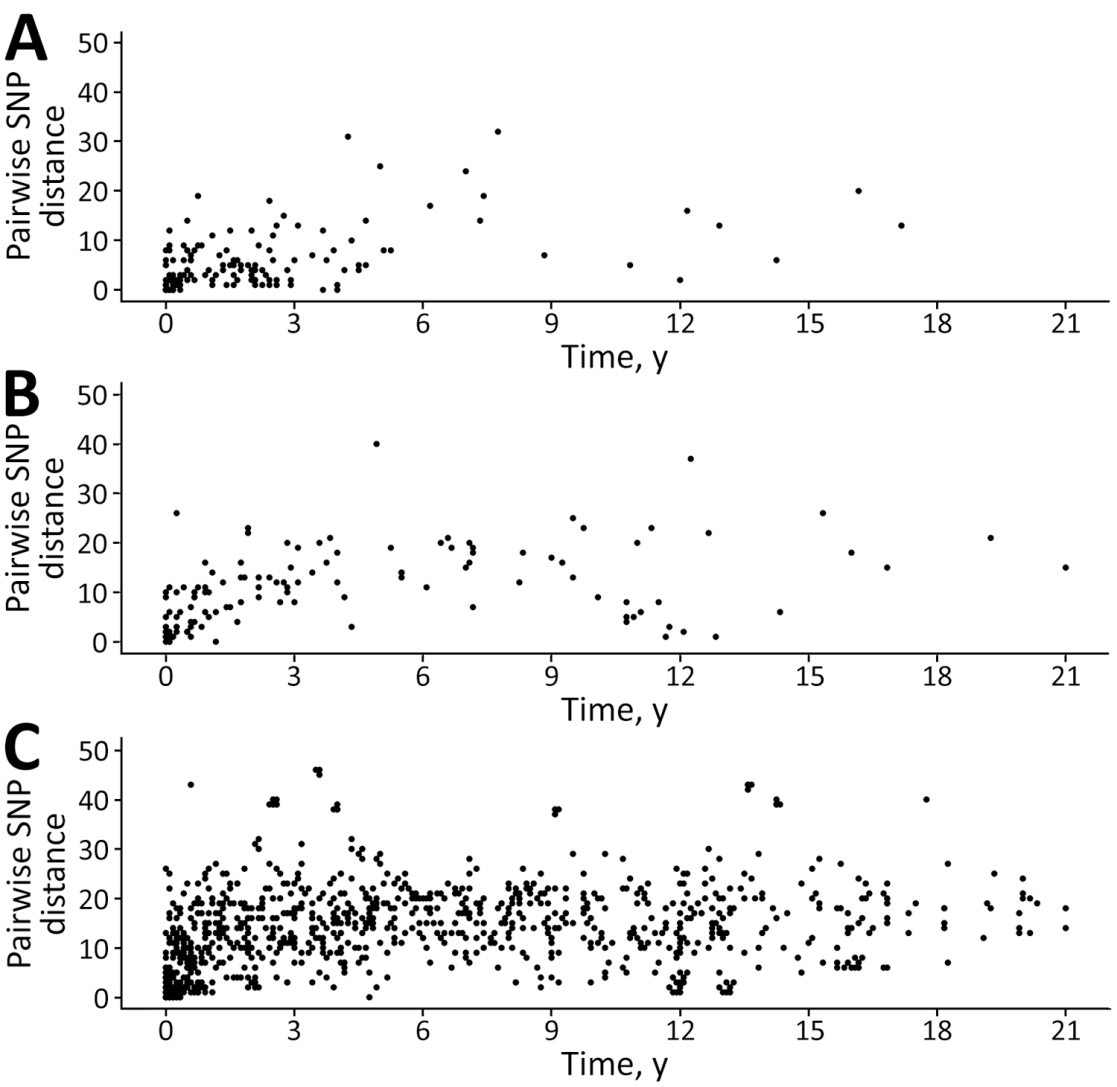
Pairwise SNP distances observed among 321 clinical isolates in clusters in retrospective analysis of historical *Listeria monocytogenes* clinical isolates, New York, USA, 2000–2021. Each data point represents a pair of isolates from the same county (A), contiguous counties (B), or noncontiguous counties (C). The timespan corresponds to the interval between each isolate pair within clusters in years with a month (1/12 of a year) as the minimum measurable unit. If the clinical isolates in a cluster were collected within the same month of the same year, the timespan for the cluster is 0. SNP, single-nucleotide polymorphism.

On the basis of the highest SNP distance within each cluster, we applied arbitrary SNP thresholds: highly related (0–10 SNP differences) and closely related (11–20 SNP differences). More than half (61%, 52/85) of New York clusters had highly related isolates (Appendix Table 1), consisting of 46 small, 5 medium, and 1 large cluster. Although the maximum SNP distances in a cluster were not statistically different between clusters classified as same and contiguous counties (β_cluster_ −0.40 [95% CI −0.88 to 0.09]; p = 0.10) compared with noncontiguous counties (β_cluster_ −0.10 [95% CI −0.58 to 0.41]; p = 0.69), 89% (16/18) of clusters from the same county presented high genetic relatedness (maximum SNP distance ≤10 SNPs) (Figure 3). Conversely, only 60% (9/15) of clusters from contiguous counties and 52% (27/52) of clusters from noncontiguous counties exhibited high relatedness, suggesting that localized clusters tend to be associated with more genetically related isolates. In addition, the within-cluster pairwise SNP distance between isolates from same and contiguous counties (Figure 4, panels A, B) were significantly lower (β_pairwise_ −3.68 [95% CI −4.72 to −2.64]; p = 0.03) than the pairwise SNP distance between isolates from noncontiguous counties (β_pairwise_ −1.05 [95% CI −2.00 to −0.09]; p<0.001) (Figure 4, panel C). Both the maximum SNP distance (β_cluster_ 0.005 [95% CI 0.001–0.009]; p = 0.003) and the within-cluster pairwise SNP distances between isolates (β_pairwise_ 0.015 [95% CI 0.01–0.02]; p<0.001) were significantly positively associated with the timespan between isolates.

In addition to SNP-based distances, we assessed the relationship between pairwise SNP distances and cgMLST allele distances across all isolate pairs for isolates in a given cluster. We observed a strong positive correlation between SNP and allele distances (Pearson correlation coefficient 0.96) (Appendix Figure 1), indicating that cgMLST allele distances closely mirror SNP distances; SNP distances ranged from 0 to 67 and cgMLST allele distances ranged from 0 to 32. We observed a strong linear relationship between SNP and allele distances across the range of 0 to 67 SNPs. Specifically, the slope of the regression line was ≈0.5, indicating 1 allele difference for every 2 SNP differences. Therefore, the SNP threshold of 20 SNP differences used here was equivalent to a difference of 10 cgMLST alleles. In general, isolates considered to be highly related (i.e., <10 SNPs) showed <5 allele differences between each other.

### Isolates with New York–Specific Outbreak Codes

In a listeriosis multistate outbreak investigation, CDC PulseNet assigns outbreak codes to clusters with >3 clinical isolates collected within 120 days, with >2 clinical isolates differing by <5 cgMLST alleles. Seven isolates with outbreak codes that were previously assigned to only 1 clinical isolate in our dataset were assigned to New York clusters, suggesting that more cases might have been part of those outbreaks but were not previously identified. Clusters included isolates with 22 different outbreak codes, including 5 New York–specific and 17 multistate codes (Appendix Table 4). Five New York outbreak codes were linked to 1 medium and 3 small clusters; 2 codes were assigned to isolates in the same medium cluster (NY-cluster 18). The multistate outbreak codes were linked to 5 small, 3 medium, and 2 large clusters; the 2 large clusters were linked with 2 multistate outbreak codes (NY-cluster 84) and 7 multistate codes (cluster 85) (i.e., isolates obtained at similar times were assigned the same code, whereas genetically related isolates obtained at different times were assigned different outbreak codes) (Appendix Table 4).

All 4 clusters linked with New York outbreak codes included highly related isolates with SNP differences ranging from 0 to 6 SNPs (median 2 SNPs) (Appendix Table 1). Of those clusters with New York outbreak codes, 3 were also short-lasting (0–1 month); the other cluster with a New York outbreak code spanned 4.5 years (NY-cluster 18). Of the 4 clusters with New York outbreak codes, 2 included isolates from the same county (including NY-cluster 18), whereas the other 2 clusters included isolates from noncontiguous counties. Hence, no clusters with New York–specific outbreak codes included isolates from different counties or with cases >1 month apart (Appendix Table 1).

### Relationship with Nonclinical Isolates

**Figure 5:**
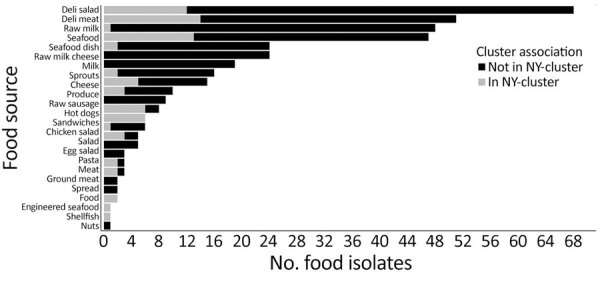
Number of food isolates obtained by food category in retrospective analysis of historical *Listeria monocytogenes* clinical isolates, New York, USA, 2000–2021. Some categories reflect limited metadata detail: food indicates unspecified food products, spread includes unspecified spreadable foods (e.g., cheese or other dips), and seafood dish includes prepared seafood dishes, whereas seafood indicates raw or unspecified seafood items.

A total of 307 nonclinical isolates (78 food, 4 animal, and 225 environmental) differed by <50 SNP differences from >1 New York clinical isolate in 37 clusters (Appendix Table 5). The most common types of food isolates within clusters were deli meats (n = 18) and fish-related products (n = 15) (Figure 5). Fourteen clusters had >2 clinical and nonclinical isolates obtained from the same county (Appendix Table 6). Seventeen clusters included nonclinical isolates highly related (SNP distances <10) to >1 clinical isolate (Appendix Table 6); 13 were small or medium.

## Discussion

Before WGS became the major subtyping method applied to public health, US multistate listeriosis outbreaks were considered rare (<10 outbreaks/year), short-lasting (usually <1 year in length), and medium to large (most outbreaks had >10 cases) (*2*,*12*). Since WGS was implemented in the United States in 2013 (*4*), more outbreaks (>10 per year) have been identified, many of which last >1 year and involve fewer cases (most with <10 cases) (*2*). We used a collection of >1,000 clinical *L. monocytogenes* isolates obtained in New York during 2000–2021 to assess temporal and geographic distribution of clusters of closely related isolates and the patterns associated with clusters that included isolates with New York cluster codes and outbreak codes. Our results show that New York clusters were generally small (<3 cases per cluster) and long-lasting (median timespan >2 years) and commonly involved isolates from the same or contiguous counties (≈40%). Furthermore, NYSDOH-assigned cluster codes were significantly associated with clusters with isolates exclusively obtained after 2014, when WGS began to be used alongside PFGE as a subtyping method for *L. monocytogenes* clinical isolates, suggesting that WGS has greatly improved the identification of *L. monocytogenes* clusters. In addition, New York outbreak codes were linked to clusters, including highly related (<10 SNP differences) clinical isolates that occurred within 2 months of each other or originated from the same county. Of note, 37 clusters, including 23 (62%) clusters with 2 or 3 cases only, had >1 environmental or food isolate related (within <50 SNPs) to >1 clinical isolate, suggesting that a possible source could be linked to those clusters.

Our findings indicate that a substantial number of listeriosis cases represent small, long-lasting clusters. Surveillance systems capable of detecting such clusters could enhance prevention efforts. Most clusters in our study were small (2–3 clinical isolates) and spanned >2 years. Similarly, a previous study analyzed *L. monocytogenes* population structure of clinical isolates in New York during 2000–2021 and found 38 epidemiologically linked clusters (including >3 clinical isolates) showing multiyear persistence of 20 identified clusters (*13*). Likewise, studies in the Netherlands (*14*,*15*), Germany (*16*), United Kingdom (*17*), and Italy (*18*,*19*) using WGS data on historical *L. monocytogenes* clinical isolates showed small clusters (i.e., 2–3 cases) scattered over time. The observation that many small clusters were scattered over long periods suggests that investigations of *L. monocytogenes* clusters involving as few as 2 cases, when the isolates are separated by <20 SNPs, even if the cases occurred >1 year apart, might be valuable and could contribute to improved prevention.

The observation of many clusters scattered over long periods might suggest that these clusters might be caused by reoccurring, emerging, or persisting (REP) *L. monocytogenes* strains. CDC defines these strains as reoccurring (causing repeated cases over an extended period but not causing continuing cases), emerging (strains start to cause an increasing number of cases), or persisting (strains that consistently cause illnesses over extended periods) (*20*). In addition to strains of *Salmonella enterica* (*21*) and *Escherichia coli* O157:H7 (*22*), *L. monocytogenes* also includes REP strains (https://www.cdc.gov/foodborne-outbreaks/php/rep-surveillance/index.html). Although none of the 85 clusters identified included confirmed *L. monocytogenes* REP strains, our findings indicate that several *L. monocytogenes* strains in New York might represent local or previously unidentified REP-like strains. For example, the largest cluster identified in this study included >1 isolate reported in each year during 2000–2021, except for the years 2003, 2014, 2015 and 2019; this cluster could, thus, be linked to a persisting *L. monocytogenes* strain (Appendix Figure 2). All large and most medium clusters showed evidence for persistence similar to that reported as characteristic for REP strains; more specifically, most medium and all large clusters included clinical isolates obtained >2 years apart. Identifying REP strains can be key for outbreak source attribution. According to CDC, REP strain designation considers the number of cases and rising case numbers associated with the strain, and how frequent or large the outbreaks associated with this strain are. REP strains might show multidrug resistance, increased virulence, or increased transmissibility than non-REP strains (*20*). Because we did not characterize strains comprehensively, we refer to some of the clusters persisting >2 years as REP-like strains. The many REP-like strains identified in this and previous studies (*13*–*19*) is not necessarily surprising, given *L. monocytogenes*’s ability to persist for many years in food-associated environments (*1*,*23*–*31*) and to recontaminate finished products after many years (*26*,*32*–*35*).

To further understand genetic relatedness within clusters, we compared pairwise SNP and cgMLST allele distances. The strong correlation between SNP and cgMLST allele distances (Pearson correlation coefficient 0.96) supported the concordance between the 2 high-resolution typing methods (*36*). However, the broader numerical range of SNP distances (0–67) compared with cgMLST allele distances (0–32) indicates that cgMLST might reveal less genetic variation; that difference is likely because of SNP differences that fall in genes not included in the cgMLST scheme or that fall in intergenic regions. Hence, for the 1,748-gene cgMLST scheme, a threshold of <7 allele difference has been suggested for clustering related isolates (*37*), which closely aligns with the <10 allele difference we observed for isolates <20 SNPs apart. Although cgMLST offers standardization and ease of comparison for routine surveillance (e.g., through PulseNet), our results highlight the importance of SNP analysis offering finer discrimination and more precise cluster boundaries, supporting its use as a complementary method for validating or refining cgMLST-based clusters (*36*,*38*).

Although linking nonclinical isolates to clinical clusters might provide valuable clues for investigating clusters and identifying sources, traceback and epidemiologic evidence remain essential to link clusters to specific sources. That process can be particularly challenging for small or temporally dispersed clusters, where obtaining conclusive evidence is often difficult. In our analysis, a <50 SNP threshold to link nonclinical isolates with clusters was intended as an exploratory approach. That threshold is not intended to confirm that isolates are part of an outbreak linked to a common source but rather to identify potentially related isolates that can guide hypotheses and prioritize targets for epidemiologic investigations. Several small and medium clusters included food and environmental isolates highly related to clinical isolates from the same county, even when collected years apart. This finding could suggest local common sources and persistent contamination in local small processors, farms, retail establishments, or private (e.g., residential) and public (e.g., schools, hospitals, restaurants) kitchens (*1*). Similarly, long-lasting listeriosis outbreaks linked to local settings have been previously reported, including a hospital-linked outbreak in Finland consisting of 6 listeriosis cases during 2015–2019 (*39*) and a food-service-linked outbreak in the United States consisting of 15 listeriosis cases during 2014–2017 (*40*). Those results support the importance of comprehensive data collection and analysis of environmental and food isolates, which can support Sample-Initiated Retrospective Outbreak Investigations and help identify potential sources and initiate inspections to determine the specific source of outbreaks (*4*).

Our findings indicate that WGS of historical *L. monocytogenes* isolates could contribute to identifying long-lasting outbreaks previously recognized as sporadic cases or as smaller, independent, acute outbreaks. Only 4 clusters were linked to New York outbreak codes and just 10 clusters were linked to multistate outbreak codes, despite 25 clusters containing highly related clinical isolates (<10 SNPs) from the same or contiguous counties. Although clusters containing closely related clinical isolates (<20 SNPs) obtained during 2000–2013 might have been overlooked because of PFGE’s lower resolution (*2*,*4*), 20 clusters included isolates that were exclusively obtained after 2013. Clusters without a linked outbreak code were usually small (2–3 clinical isolates) and had clinical isolates that were closely but not highly related or that were obtained >1 year apart. Since the advent of WGS as a subtyping tool in public health, long-lasting listeriosis outbreaks have been more commonly recognized (e.g., outbreaks linked to queso fresco and cotija cheese in 2014–2024 [*41*], enoki mushrooms in 2016–2019 [*42*], leafy greens in 2018–2023 [*43*], packaged salads in 2015–2022 [*44*], and deli meats and cheeses in 2016–2019 [*45*]), suggesting that at least some outbreaks initially considered to be short-lasting might actually represent larger, long-lasting outbreaks. This possibility is supported by clusters 18, 84, and 85, which included clinical isolates that were linked to multiple short-spanning outbreaks (i.e., the isolates in those clusters were linked to multiple PulseNet-assigned outbreak codes). Moreover, cases years apart that included closely related isolates tended not to be investigated as part of an outbreak. For example, 15 clusters included highly related (<10 SNPs) clinical isolates that were obtained >2 years apart and were not assigned outbreak codes; only 1 of the 15 clusters included >3 isolates. This possibility is particularly likely for clusters with closely related isolates from cases that occurred when different subtyping methods (i.e., PFGE and WGS) were being used. Although both PFGE and WGS were used during 2014–2017, cases before 2014 were analyzed using PFGE, which is less sensitive for detecting outbreaks (*2*), whereas cases after 2017 were only analyzed by WGS.

In conclusion, using a data-driven approach, we demonstrated that outbreak investigations often prioritize temporally tight clusters of highly related isolates, whereas small clusters that are spread across time but are from the same or contiguous counties are frequently overlooked. Our results emphasize the importance of comparing new and historical *L. monocytogenes* clinical isolates using WGS because even small listeriosis outbreaks could span a long period of time because of the bacteria’s long-term persistence in food-associated environments. Moreover, small and medium clusters including food and environmental isolates from the same counties as the clinical isolates could also indicate that the source might be a small local food-associated environment (e.g., local ice cream manufacturer, packing house, restaurant, hospital). Our study also highlights the importance of performing routine environmental and food surveillance to enable public health and regulatory agencies to identify sources prone to persistence and take necessary control measures to mitigate further risk.

Appendix Additional information about retrospective analysis of historical *Listeria monocytogenes* clinical isolates, New York, USA, 2000–2021
