# Supplementary material for: Retrospective Analysis of Historical Listeria monocytogenes Clinical Isolates, New York, USA, 2000–2021
Source: Emerg Infect Dis. 2025 Oct;31(10):1883–92. doi: 10.3201/eid3110.241876 (PMC12483022; doi:10.3201/eid3110.241876)
Supplement: Appendix — Additional information about retrospective analysis of historical Listeria monocytogenes clinical isolates, New York, USA, 2000–2021 [file 24-1876-Techapp-s1.pdf]

Article DOI: https://doi.org/10.3201/eid3110.241876

EID cannot ensure accessibility for supplementary materials supplied by authors. Readers who have difficulty accessing supplementary content should contact the authors for assistance.

Retrospective Analysis of Historical *Listeria monocytogenes* Clinical Isolates, New York, USA, 2000–2021

Appendix

Materials and Methods

Whole genome sequencing.

A total of 957 Lm clinical isolates obtained by NYSDOH between January 2000 and September 2021 and 89 clinical isolates obtained by the New York City Department of Health and Mental Hygiene (NYCDOHMH) between January 2000 and 2004 (isolation month unknown) were whole genome sequenced with an Illumina Next-Seq 500 instrument (Illumina, San Diego, CA). NGS Libraries were prepared using the Illumina DNA Prep kit (formerly Nextera Flex, part number 20060059), using a modified, quarter scale protocol as described by Dickinson et al. (2024) (*1*). In addition, 802 non-clinical isolates (i.e., environmental and food isolates) obtained by the New York State Department of Agriculture and Markets (NYSAGM) between January 2000 and December 2021 were whole genome sequenced using an Illumina MiSeq instrument (Illumina, San Diego, CA). Libraries were prepared using either Illumina Nextera XT or Illumina DNA Prep kits. Additional WGS data for 523 non-clinical isolates collected between 2000 (isolation month unknown) and May 2020 were obtained from previous studies (*2*,*3*). Whole genome sequencing (WGS) data were submitted to PulseNet and National Center for Biotechnology Information (NCBI) as part of (i) routine surveillance activities carried out by the NYSDOH and NYSAGM, (ii) sequencing of historical isolates from NYCDOHMH NYSDOH and NYSAGM, and (iii) previous research projects from our group (*2*,*3*). The publicly available WGS data were assigned the following BioProject accession numbers: PRJNA212117,

PRJNA215355, PRJNA302599, PRJNA351287, PRJNA483181, PRJNA514286, PRJNA664209, PRJNA245909, PRJNA395587, PRJNA514285, PRJNA561882, PRJNA512470, PRJNA514288. From NCBI, the data was added to the Pathogen Detection (PD) browser. NCBI PD assigns isolates to SNP clusters using criteria detailed in Stevens et al. (*4*). Pairwise single nucleotide polymorphisms (SNPs) were identified between isolates within the same SNP cluster and the data are stored in the NCBI PD File Transfer Protocol (FTP) site, which provides the analyses that have been completed on sequence reads uploaded to NCBI (*5*,*6*).

Listeria monocytogenes **clinical isolate metadata and data filtration.**

Isolates' metadata and genetic relatedness (i.e., the pairwise SNP distances) were downloaded from the NCBI PD database (https://www.ncbi.nlm.nih.gov/pathogens/; Accession date: October 4, 2023). Additional metadata (i.e., collection date, county-level geolocation, detailed source description, cluster codes, and outbreak codes) were available from Food Microbe Tracker (FMT; a web-based tool to store information regarding food-associated microorganisms), NYSDOH, and NYSAGM for selected isolates. Pathogen Detection Target (PDT) accession numbers, assigned by NCBI PD to each genome assembly uploaded to NCBI, were used as isolate identifiers. When isolates showed distinct PDT numbers but the same strain IDs (e.g., Cornell University Food Safety Laboratory ID, PulseNet ID), suggesting multiple sequences of the same isolates, only one instance was kept. Isolates obtained at most 2 months apart, and from the same or contiguous counties, and differing by <5 SNPs were further reviewed to identify possible isolates obtained from the same patient or mother-child pairs (i.e., representing vertical transmission). Forty clinical isolates representing mother-child pairs or isolates from the same patient were excluded from the study (i.e., only one isolate per instance was kept).

Clinical isolate clustering and identification of non-clinical isolates in NY-clusters.

Genetic relatedness of clinical isolates was assessed based on the pairwise SNP distances obtained from NCBI PD. For clustering the isolates into "NY-clusters," a threshold of ≤20 SNPs was used in a single-linkage clustering approach (i.e., to be added to a NY-cluster, one clinical isolate needs to show a SNP distance of ≤20 SNPs to at least one of the other clinical isolates in that NY-cluster). NY non-clinical isolates differing by ≤50 SNPs from clinical isolates in a NY-cluster were added to the clinical isolate NY-cluster. A threshold of ≤20 SNPs for clinical

isolates was selected based on the maximum pairwise SNP distances typically observed in foodborne outbreak investigations (*7–9*) and to accommodate higher genetic diversity that could be expected from isolates obtained years apart. A less stringent threshold of ≤50 SNPs was used between clinical and non-clinical isolates for initial screening purposes, consistent with previous studies that used similar criteria to identify strains potentially persisting within the same facility (*10*).

NY-clusters were classified as (i) "same" county, when all isolates in a NY-cluster were obtained from the same county, (ii) "contiguous" counties, when at least two isolates were obtained from counties that are bordering each other, and no isolates are from noncontiguous counties and (iii) "noncontiguous" counties, when at least two isolates in the NY-cluster were obtained from counties that do not share borders.

Core-genome multilocus sequence typing (cgMLST).

cgMLST analysis was performed using PulseNet 2.0. Raw sequence files were uploaded to PulseNet 2.0, which automatically performed read quality control and taxonomic assignment using the Metagenomic Intra-species Diversity Analysis System (MIDAS) (*11*). Next, SPAdes generated assemblies and critical sequence quality metrics were automatically assessed as either passing or failing (*12*). Except for eight isolates, all other Lm assemblies passed the following quality control criteria: (i) average nucleotide identity (ANI) percent aligned >75%, (ii) ANI score >92%, (iii) average de novo coverage >20X, (iv) average quality score (Q score) >30, (v) assembly length (2.8 Mbp – 3.19 Mbp), (vi) MIDAS secondary coverage <1.0%, and (vii) percent core genome allele called >95%. Following quality control, assembly-based allele calling was compared against an internal Lm allele database of 1,748 cgMLST loci (*13*).

Statistical analysis.

All analyses were performed in R version 4.2.1. The generalized least square (GLS) model (R package NMLE (*14*)) was used to assess a significant association between pairwise SNP distances and the time interval for each isolate pair based on whether the isolates are from the same, contiguous, or noncontiguous counties. The GLS model was implemented with a modification of Clarke's maximum likelihood population effects (MLPE) model (*15*) using the corMLPE tool (*16*). To assess whether the maximum SNP distance observed between a pair of cases in a NY-cluster could be predicted from the corresponding time interval between these

cases, a generalized linear model (GLM) with a negative binomial distribution was used (*17*). The estimated mean differences in pairwise SNP distances ($ß_{pairwise}$) and maximum SNP distance between clusters ($ß_{cluster}$) were reported. Chi-square test was used to test the association between NY-cluster size and the regions where the isolates in NY-clusters were collected from. All statistical tests were performed at a significance level of 0.05.

References

1. Dickinson MC, Wirth SE, Baker D, Kidney AM, Mitchell KK, Nazarian EJ, et al. Implementation of a high-throughput whole genome sequencing approach with the goal of maximizing efficiency and cost effectiveness to improve public health. Microbiol Spectr. 2024;12:e0388523. PubMed https://doi.org/10.1128/spectrum.03885-23

2. Jagadeesan B, Baert L, Wiedmann M, Orsi RH. Comparative analysis of tools and approaches for source tracking *Listeria monocytogenes* in a food facility using whole-genome sequence data. Front Microbiol. 2019;10:947. PubMed https://doi.org/10.3389/fmicb.2019.00947

3. Stasiewicz MJ, Oliver HF, Wiedmann M, den Bakker HC. Whole-genome sequencing allows for improved identification of persistent *Listeria monocytogenes* in food-associated environments. Appl Environ Microbiol. 2015;81:6024–37. PubMed https://doi.org/10.1128/AEM.01049-15

4. Stevens EL, Carleton HA, Beal J, Tillman GE, Lindsey RL, Lauer AC, et al. Use of whole genome sequencing by the Federal Interagency Collaboration for genomics for food and feed safety in the United States. J Food Prot. 2022;85:755–72. PubMed https://doi.org/10.4315/JFP-21-437

5. National Center for Biotechnology Information. Pathogen detection help document beta release [cited 2024 Apr 26]. https://www.ncbi.nlm.nih.gov/pathogens/pathogens_help/

6. National Center for Biotechnology Information (NCBI). Methods description for ftp://ncbi.nlm.nih.gov/pathogen/ [cited 2024 Apr 26]. https://ftp.ncbi.nlm.nih.gov/pathogen/Methods.txt

7. Pightling AW, Pettengill JB, Luo Y, Baugher JD, Rand H, Strain E. Interpreting whole-genome sequence analyses of foodborne bacteria for regulatory applications and outbreak investigations. Front Microbiol. 2018;9:1482. PubMed https://doi.org/10.3389/fmicb.2018.01482

8. Chen Y, Gonzalez-Escalona N, Hammack TS, Allard MW, Strain EA, Brown EW. Core genome multilocus sequence typing for identification of globally distributed clonal groups and

differentiation of outbreak strains of *Listeria monocytogenes.* Appl Environ Microbiol. 2016;82:6258–72. PubMed https://doi.org/10.1128/AEM.01532-16

9. Jackson BR, Tarr C, Strain E, Jackson KA, Conrad A, Carleton H, et al. Implementation of nationwide real-time whole-genome sequencing to enhance listeriosis outbreak detection and investigation. Clin Infect Dis. 2016;63:380–6. PubMed https://doi.org/10.1093/cid/ciw242

10. Pettengill JB, Rand H, Wang SS, Kautter D, Pightling A, Wang Y. Transient and resident pathogens: intra-facility genetic diversity of *Listeria monocytogenes* and *Salmonella* from food production environments. PLoS One. 2022;17:e0268470. **PMID 36048885**

11. Zhao C, Smith BJ, Dimitrov B. MIDAS version 3 [cited 2025 May 20]. https://zenodo.org/records/6967726

12. Bankevich A, Nurk S, Antipov D, Gurevich AA, Dvorkin M, Kulikov AS, et al. SPAdes: a new genome assembly algorithm and its applications to single-cell sequencing. J Comput Biol. 2012;19:455–77. PubMed https://doi.org/10.1089/cmb.2012.0021

13. Moura A, Criscuolo A, Pouseele H, Maury MM, Leclercq A, Tarr C, et al. Whole genome-based population biology and epidemiological surveillance of *Listeria monocytogenes.* Nat Microbiol. 2016;2:16185. PubMed https://doi.org/10.1038/nmicrobiol.2016.185

14. Pinheiro J, Bates D, DebRoy S, Sarkar D. nlme: Linear and nonlinear mixed effects models. R package version 3.1–164 [cited 2024 Apr 22]. https://cran.r-project.org/web/packages/nlme

15. Clarke RT, Rothery P, Raybould AF. Confidence limits for regression relationships between distance matrices: estimating gene flow with distance. J Agric Biol Environ Stat. 2002;7:361–72. https://doi.org/10.1198/108571102320

16. Pope N. GitHub - nspope:corMLPE: a corStruct class for nlme, implementation of Clarke's maximum likelihood population effects model [cited 2024 Apr 22]. https://github.com/nspope/corMLPE?tab=readme-ov-file

17. Marschner IC. glm2: fitting generalized linear models with convergence problems. R J. 2011;3:12–5. https://doi.org/10.32614/RJ-2011-012

Appendix Table 1. Characteristics of the 85 NY-clusters identified in New York State between 2000 and 2021

Cluster IDs*	No. of clinical isolates	Isolation date of the first isolate	Isolation date of the last isolate	No. of counties	SNP distance range†	cgMLST allele distance range†	Relatedness§	No. of isolates previously assigned a cluster code by NYSDOH	Type of outbreak if NY-cluster includes isolates with PulseNet outbreak code
Same county (n = 18)									
Small clusters (n = 17)									
1	2	Jul 2018	Jul 2018	1	6	2	highly-related	2/2	multistate
2	2	Sep 2020	Oct 2020	1	0	0	highly-related	0/2	multistate
3	2	Oct 2015	Nov 2015	1	2	1	highly-related	2/2	NY-specific
4	2	Jul 2019	Aug 2019	1	12	6	closely-related	2/2	none
5	2	Mar 2004	May 2004	1	0	0	highly-related	0/2	none
6	2	Jul 2009	Mar 2010	1	2	2	highly-related	0/2	none
7	2	Jul 2007	Jul 2008	1	4	1	highly-related	0/2	none
8	2	Aug 2009	Nov 2010	1	7	3	highly-related	0/2	none
9	2	Oct 2011	May 2013	1	3	1	highly-related	0/2	none
10	2	Sep 2002	Nov 2004	1	9	5	highly-related	0/2	none
11	2	Jul 2017	Dec 2019	1	8	NA ‡	highly-related	2/2	none
12	2	Jan 2017	Jul 2019	1	11	6	closely-related	2/2	none
13	2	Aug 2014	Jul 2018	1	8	3	highly-related	2/2	none
14	2	Dec 2012	Aug 2017	1	5	2	highly-related	0/2	none
15	3	Jan 2010	Mar 2010	1	0	0–1	highly-related	0/3	none
16	3	Oct 2015	May 2017	1	2–8	0–2	highly-related	0/3	none
17	3	Oct 2011	Nov 2016	1	4–8	2–3	highly-related	0/3	none
Medium clusters (n = 1)									
18	9	Jun 2014	Dec 2018	1	0–6	0	highly-related	8/9 ¶	NY-specific (2 codes)
Contiguous counties (n = 15)									
Small clusters (n = 12)									
19	2	Dec 2013	Jan 2014	2	6	3	highly-related	0/2	none
20	2	Jan 2006	Apr 2006	2	2	0	highly-related	0/2	none
21	2	Jan 2000	Apr 2000	2	5	3	highly-related	0/2	none
22	2	Apr 2020	Oct 2020	2	2	2	highly-related	2/2	none
23	2	Aug 2014	Aug 2015	2	5	3	highly-related	2/2	none
24	2	Feb 2006	Mar 2009	2	12	4	closely-related	0/2	none
25	2	Nov 2006	Nov 2013	2	15	8	closely-related	0/2	none
26	3	Jul 2005	Aug 2005	2	0	0	highly-related	2/3	none
27	3	Jul 2012	Dec 2014	2	2–18	2	closely-related	0/3	none
28	3	Sep 2015	Sep 2018	2	4–8	0 ‡	highly-related	0/3	none
29	3	Aug 2010	May 2021	2	0–5	0–4	highly-related	0/3	none
30	3	Sep 2000	Nov 2020	2	7–23	1–10	closely-related	1/3	none
Medium clusters (n = 3)									
31	4	Jul 2000	Sep 2002	3	8–12	4–7	closely-related	0/4	none
32	5	Jun 2000	Dec 2003	4	5–15	3–6	closely-related	0/5	none
33	5	Aug 2004	Dec 2018	2	0–7	0–3 ‡	highly-related	3/5	none
Noncontiguous counties (n = 52)									
Small clusters (n = 38)									
34	2	Sep 2019	Sep 2019	2	0	0	highly-related	2/2	NY-specific
35	2	Nov 2002	Nov 2002	2	6	3	highly-related	0/2	none
36	2	Oct 2009	Nov 2009	2	0	0	highly-related	0/2	none
37	2	Oct 2009	Nov 2009	2	0	0	highly-related	0/2	none

Cluster IDs*	No. of clinical isolates	Isolation date of the first isolate	Isolation date of the last isolate	No. of counties	SNP distance range†	cgMLST allele distance range†	Relatedness§	No. of isolates previously assigned a cluster code by NYSDOH	Type of outbreak if NY-cluster includes isolates with PulseNet outbreak code
38	2	Sep 2009	Oct 2009	2	0	0	highly-related	0/2	none
39	2	Aug 2015	Sep 2015	2	1	0	highly-related	0/2	multistate
40	2	Aug 2019	Sep 2019	2	12	6	closely-related	2/2	none
41	2	Aug 2003	Nov 2003	2	0	0	highly-related	0/2	none
42	2	May 2001	Sep 2001	2	0	1	highly-related	0/2	none
43	2	Aug 2001	Mar 2002	2	1	0	highly-related	0/2	none
44	2	Jun 2003	Mar 2004	2	10	4	highly-related	0/2	none
45	2	Feb 2004	Dec 2004	2	17	9	closely-related	0/2	none
46	2	Aug 2002	Sep 2003	2	1	1	highly-related	0/2	none
47	2	Sep 2002	Oct 2003	2	2	1	highly-related	0/2	none
48	2	Dec 2017	Feb 2019	2	15	7	closely-related	1/2	none
49	2	Oct 2018	Jan 2020	2	10	6	highly-related	2/2	none
50	2	Feb 2016	Aug 2017	2	11	8	closely-related	0/2	none
51	2	Dec 2012	Jun 2016	2	12	5	closely-related	2/2	multistate
52	2	Nov 2010	Oct 2014	2	11	NA ‡	closely-related	0/2	none
53	2	Oct 2000	Nov 2004	2	13	6	closely-related	0/2	none
54	2	Jun 2002	Sep 2006	2	16	11	closely-related	0/2	none
55	2	Oct 2000	Jul 2003	2	0	0	highly-related	0/2	none
56	2	Sep 2007	Jun 2012	2	5	2	highly-related	0/2	none
57	2	Jul 2008	Sep 2013	2	14	5	closely-related	0/2	none
58	2	Oct 2011	May 2017	2	13	5	closely-related	1/2	multistate
59	2	Dec 2010	Sep 2017	2	9	3	highly-related	0/2	none
60	2	Apr 2011	May 2018	2	17	10	closely-related	1/2	none
61	2	Aug 2003	Jul 2012	2	14	7	closely-related	0/2	none
62	2	Aug 2006	Jul 2016	2	10	7	highly-related	0/2	none
63	2	Jan 2009	Oct 2019	2	8	4	highly-related	0/2	none
64	2	Aug 2000	Jul 2013	2	5	3	highly-related	0/2	none
65	3	Aug 2018	Aug 2018	3	2	0–1	highly-related	3/3	NY-specific
66	3	Jul 2006	Aug 2006	3	0–1	0–1	highly-related	0/3	none
67	3	Sep 2002	Jan 2003	3	0–1	0–1	highly-related	0/3	none
68	3	Oct 2000	Jan 2003	3	4–18	3–13	closely-related	0/3	none
69	3	Oct 2016	Apr 2019	3	7–16	2–9	closely-related	3/3	none
70	3	Oct 2003	Feb 2013	3	4–22	3–12	closely-related	0/3	none
71	3	Jul 2005	Oct 2015	3	3–4	2 ‡	highly-related	0/3	none
Medium clusters (n = 10)									
72	4	Oct 2000	Dec 2001	3	2–8	1–4	highly-related	0/4	none
73	4	Oct 2002	Nov 2013	3	8–22	3–12	closely-related	0/4	multistate
74	4	Jul 2006	May 2020	2	6–19	2–11	closely-related	0/4	none
75	5	Mar 2009	May 2010	4	1–6	0–4	highly-related	0/5	none
76	6	Aug 2001	Feb 2015	6	12–25	3–13 ‡	closely-related	1/6	none
77	6	Dec 2004	Apr 2020	4	3–11	2–5	closely-related	0/6	none
78	7	Mar 2001	Oct 2005	5	1–32	0–14 ‡	closely-related	0/7	none
79	8	Aug 2011	Jun 2021	7	1–23	1–12 ‡	closely-related	5/8	none
80	8	May 2002	Feb 2020	6	1–46	0–26	closely-related	6/8 ¶	multistate
81	9	Jul 2001	Oct 2019	6	0–40	0–24	highly-related	0/9	multistate
Large clusters (n = 4)									

Cluster IDs*	No. of clinical isolates	Isolation date of the first isolate	Isolation date of the last isolate	No. of counties	SNP distance range†	cgMLST allele distance range†	Relatedness§	No. of isolates previously assigned a cluster code by NYSDOH	Type of outbreak if NY-cluster includes isolates with PulseNet outbreak code
82	13	Jan 2002	Jun 2018	10	0–22	0–11	closely-related	1/13	none
83	19	Nov 2000	Sep 2016	8	0–18	0–9	closely-related	0/19	none
84	23	Nov 2001	Sep 2004	10	0–10	0–5	highly-related	0/23	multistate (2 codes)
85	33	Sep 2000	Sep 2021	24	0–31	1–17	closely-related	30/33	multistate (7 codes)

* Cluster IDs 1 to 85 were assigned to NY-clusters identified in this study.

† Single Nucleotide Polymorphism (SNP) or allele distances between clinical isolates in the NY-clusters. SNP or allele distances given in ranges indicate the lowest and highest SNP or allele distances in NY-clusters with 3 or more clinical cases.

‡ One or more isolates in these NY-clusters did not pass the PulseNet2.0 quality criteria, and, therefore, could not be analyzed using the cgMLST scheme.

§ “Highly-related” means that the highest number of SNP differences between the two most related isolates in the cluster is ≤10; “closely-related” means that the highest number of SNP differences between the two most closely-related isolates in the cluster is >10 but ≤20.

¶ NY-clusters 18 and 80 included isolates with 2 cluster codes. For NY-cluster 18, one cluster code included 5 isolates from 2014 and 2018, and the other cluster code included 3 isolates from 2016 and 2017. An earlier isolate from 2016 was not assigned a cluster code. For NY-cluster 80, one cluster code included 4 isolates from 2015 and 2020, and the other cluster code included 2 isolates from 2014 and 2019. Two isolates from 2002 and 2006 were not assigned a cluster code.

Appendix Table 2. The number of clinical cases by New York State counties between 2000 and 2021

County	All isolates	Isolates in NY-clusters with SNPs ≤20
NYS counties except for NYC boroughs*		
Nassau	139	41
Suffolk	129	38
Westchester	88	24
Erie	68	22
Onondaga	33	14
Monroe	49	12
Orange	30	12
Rockland	27	11
Saratoga	21	7
Broome	24	6
Schenectady	21	6
Ulster	12	6
Genesee	9	5
Rensselaer	15	5
Albany	31	4
Wayne	12	4
Chautauqua	14	3
Columbia	11	3
Dutchess	17	3
Livingston	5	3
Oneida	17	3
Ontario	12	3
Steuben	8	3
Cayuga	6	2
Cortland	6	2
Delaware	7	2
Franklin	4	2
Montgomery	6	2
Putnam	5	2
St. Lawrence	4	2
Washington	5	2
Yates	3	2
Allegany	3	1
Essex	1	1
Fulton	2	1
Greene	8	1
Jefferson	8	1
Niagara	6	1
Schuyler	2	1
Seneca	1	1
Sullivan	4	1
Tioga	6	1
Tompkins	6	1
Warren	2	1
Cattaraugus	3	-
Chemung	1	-
Chenango	7	-
Clinton	3	-
Madison	4	-
Orleans	2	-
Oswego	2	-
Otsego	3	-
Schoharie	3	-
Wyoming	1	-
Null	10	-
NYC boroughs †		
Brooklyn (Kings)	45	20
Queens	38	17
Bronx	19	11
Manhattan (New York)	8	2
Staten Island (Richmond)	10	3

*Time period covered between 2000 and 2021.
†Time period covered between 2000 and 2003. The county names for these boroughs are stated in parenthesis.
-: There are no isolates in NY-clusters with SNPs ≤20.

Appendix Table 3. Distribution of clinical isolates in NY-clusters across large, medium and small NY-clusters in New York State regions described by NYSDOH

Region*	Large NY-cluster	Medium NY-cluster	Small NY-cluster
New York City †	23	18	13
Metropolitan	36	33	66
Capital District	7	7	23
Central	8	8	16
Western	14	18	31

*Epidemiologic regions in NY described by the NYSDOH: (i) New York City: Kings, Bronx, Queens, New York, Richmond; (ii) Metropolitan area: Nassau, Suffolk, Westchester, Rockland, Putnam, Orange, Dutchess, Ulster, Sullivan; (iii) Capital District: Columbia, Greene, Delaware, Otsego, Schoharie, Albany, Rensselaer, Schenectady, Montgomery, Fulton, Saratoga, Washington, Warren, Hamilton, Essex, Franklin, Clinton; (iv) Central: Tioga, Broome, Tompkins, Cortland, Chenango, Madison, Onondaga, Cayuga, Oswego, Oneida, Herkimer, Lewis, Jefferson, St. Lawrence; (v) Western: Chautauqua, Cattaraugus, Allegany, Steuben, Chemung, Schuyler, Yates, Seneca, Ontario, Livingston, Wyoming, Erie, Genesee, Niagara, Orleans, Monroe, Wayne.
†Time period covered for this region is between 2000 and 2003.

Appendix Table 4. List of Lm outbreak investigations in New York State between 2000 and 2021

NY-cluster ID	No. of clinical cases*	First clinical isolate in NY-cluster	Last clinical isolate in NY-cluster	Geolocation connections at county level	Outbreak code†
NY-specific outbreaks					
3	2	Oct 2015	Nov 2015	Same	OutbreakCodeA
18	3	Jun 2014	Oct 2014	Same	OutbreakCodeB
18	6	Jun 2016	Dec 2018	Same	OutbreakCodeC
34	2	Sep 2019	Sep 2019	Noncontiguous	OutbreakCodeD
65	3	Aug 2018	Aug 2018	Noncontiguous	OutbreakCodeE
Multistate outbreaks					
1	2	Jul 2018	Jul 2018	Same	OutbreakCodeF
2	2	Sep 2020	Oct 2020	Same	OutbreakCodeG
39	2	Aug 2015	Sep 2015	Noncontiguous	OutbreakCodeH
51	1	Dec 2012	NA	NA	OutbreakCodeI
58	1	May 2017	NA	NA	OutbreakCodeJ
73	1	Nov 2013	NA	NA	OutbreakCodeK
80	3	Dec 2015	Jan 2016	Noncontiguous	OutbreakCodeL
81	4	Jul 2005	Jul 2005	Noncontiguous	OutbreakCodeM
84	9	Jul 2002	Sep 2002	Noncontiguous	OutbreakCodeN
84	1	Sep 2004	NA	NA	OutbreakCodeO
85	3	Jun 2013	Sep 2013	Noncontiguous	OutbreakCodeP
85	1	May 2016	NA	NA	OutbreakCodeQ
85	3	May 2004	May 2005	Noncontiguous	OutbreakCodeR
85	1	Nov 2006	NA	NA	OutbreakCodeS
85	2	Sep 2007	Oct 2007	Noncontiguous	OutbreakCodeT
85	1	Jun 2010	NA	NA	OutbreakCodeU
85	2	Mar 2012	May 2012	Noncontiguous	OutbreakCodeV

*Number of clinical isolates in NY-clusters with assigned outbreak code. NA, not applicable (i.e., the code was assigned to only one isolate).
†Outbreak codes given by CDC PulseNet were anonymized for confidentiality.

Appendix Table 5. Detailed information on the sources of non-clinical isolates in NY-clusters (non-clinical isolates differing from NY clinical isolates by ≤50 SNP differences)

Cluster ID*	No. of non-clinical isolates in NY-cluster	Duration (months)	Duration (years)	Isolation date of the first non-clinical isolate	Isolation date of the last non-clinical isolate	No. of counties	Geolocation connections of non-clinical isolates †	Counties	Geolocation connections of non-clinical isolates to clinical isolates (no. of isolates)	Types of non-clinical isolates (number of samples, county of isolation, isolation month and year, SNP distance ranges to clinical isolates in NY-cluster)	SNP distances to clinical isolates ‡
2	3	105.0	8.8	Jan.04	Oct.12	2	Noncontiguous	Kings (2); Bronx (1)	Noncontiguous	meat (1, Bronx, 1/2004, SNP: 41); seafood (1, 10/2012, SNP: 47); environmental swab sponge (1, 3/2006, SNP: 41)	41–47
3	25	167.0	13.9	Aug.03	Jul.17	5	Noncontiguous	Ulster (20); Steuben (2); Cortland (1); Onondaga (1); New York (1)	Same (20), Noncontiguous (5)	deli meat (2, 5/2016 10/2017, SNP: 1–3); hot-dogs (1, New York, 7/2002, SNP: 44–46); meat (1, 10/2017, SNP: 0–2); shellfish (1, Onondaga, 8/2003, SNP: 42–44); cold storage-dairy walk-in cooler floor (1, 7/2006, SNP: 45–47); drain raw meat (1, 10/2005, SNP:49); drain-seafood (1, 7/2006, SNP:45–47); environmental swab sponge (7, 2/2016, SNP: 1–4); sponge (10, 7/2017, 0–5)	0–49
4	4	-	-	Jun.02	2013 §	4	Noncontiguous	Tompkins (1); Tioga (1); Steuben (1); Suffolk (1)	Noncontiguous	soil (1, 2013, SNP range: 36–38); grass sampling by standing water in pasture (1, 2003, SNP range: 30–32); animal-bovine (1, 2002, SNP range: 32–34); drain-raw meat (1, 2006, SNP range: 26–28)	26–38
18	14	172.0	14.3	Oct.04	Feb.19	5	Noncontiguous	Erie (10); Bronx (1); Nassau (1); Albany (1); Kings (1)	Same (10), Noncontiguous (4)	deli meat (1, Kings, 10/2004, SNP: 25–29); environmental swab sponge (1, 10/2007, SNP: 24–28); environmental swab sponge dairy case (1, 10/2005, SNP: 27–31); environmental swab sponge floor produce area (1, 9/2005, SNP: 26–30); sponge swab/basket drain (2, 3/2019, 4/2019, SNP: 5–9); sponge swab/deli cart (1, 10/2017, SNP: 2–6); sponge swab/deli floor mat (2, 10/2017, SNP: 1–6); sponge swab/deli slicing room door (1, 10/2017, SNP: 4–8); sponge swab/drain under sink (1, 1/2019, SNP: 2–4); sponge swab/floor drain (1, 2/2019, SNP: 6–10); sponge swab/floor near sink (1, 2/2019, SNP: 2–4); sponge swab/slicer (1, 2/2019, SNP: 6–10)	1–31
21	1	0.0	0.0	Jul.03	Jul.03	1	NA	Kings	Same	deli salad	11–12
23	5	177.0	14.8	Aug.06	May.21	2 ¶	Noncontiguous (4), Unknown (1)	Sullivan (2); Suffolk (2); Unknown (1)	Contiguous (2), Noncontiguous (2), Unknown (1)	seafood dish (1, 6/2014, SNP range: 29–30); animal-avian (1, 6/2018, SNP range: 41–42); environmental swab sponge (2, 5/2021, SNP range: 38–39); drain produce area (1, 8/2006, SNP range: 31–32);	29–42
26	5	177.0	14.8	Aug.06	May.21	2 ¶	Noncontiguous (4), Unknown (1)	Sullivan (2); Suffolk (2); Unknown (1)	Same (2), Noncontiguous (2), Unknown (1)	seafood dish (1, 6/2014, SNP range: 32); animal-avian feces (1, 6/2018, SNP range: 42); environmental swab sponge (2, 5/2021, SNP range: 39); drain produce area (1, 8/2006, SNP range: 30)	30–42
27	10	48.0	4.0	Jul.02	Jul.06	8	Noncontiguous	Steuben (3); Oneida (1); Cortland (1); Queens (1), Kings (1); New York (1); Suffolk (1); Onondaga (1)	Noncontiguous	deli salad (1, Steuben, 9/2004, SNP: 42–48); hot-dogs (1, New York, 7/2002, SNP: 41–47); sandwiches (1, Kings, 8/2004, SNP: 49); sandwiches (1, Suffolk, 9/2004, SNP: 50); shellfish (1, Onondaga, 8/2004, SNP: 33–39); drain raw meat (1, 10/2005, SNP range: 40–46); cold storage-dairy walk-in cooler floor (1, 7/2006, SNP range: 36–42); dairy case (1, 7/2006, SNP range: 43–49); drain-seafood (1, 7/2006, SNP range: 36–42); sink interior (1, 9/2005, SNP: 48)	33–50

Cluster ID*	No. of non-clinical isolates in NY-cluster	Duration (months)	Duration (years)	Isolation date of the first non-clinical isolate	Isolation date of the last non-clinical isolate	No. of counties	Geolocation connections of non-clinical isolates †	Counties	Geolocation connections of non-clinical isolates to clinical isolates (no. of isolates)	Types of non-clinical isolates (number of samples, county of isolation, isolation month and year, SNP distance ranges to clinical isolates in NY-cluster)	SNP distances to clinical isolates ‡
29	3	115.0	9.6	Apr.12	Nov.21	1	Same	Kings	Noncontiguous	seafood dish (1, 4/2012, SNP: 3–4); salad (1, 5/2012, SNP: 2–3); environmental swab sponge (1, 11/2021, SNP: 9–10)	2–10
32	3	69.0	5.8	Apr.03	Jan.09	3	Noncontiguous	Otsego (2); Kings (1)	Same (1), Noncontiguous (2)	cheese (1, Otsego, 11/2003, SNP: 5–16); cheese (1, Otsego, 4/2003, SNP: 2–13); cheese (1, 1/2009, SNP range: 8–19)	2–19
33	2	0.0	0.0	Jan.19	Jan.19	1	NA	Suffolk	Same	sponge swab/under floor mat	4–8
35	2	-	-	2008 §	Sep.08	2	Noncontiguous	Bronx (1); Kings (1)	Noncontiguous	environmental swab sponge (1, 2008, SNP:26–28); sponge (1, 9/2008, SNP: 27–29)	26–29
36	3	-	-	May.02	2003 §	2	Noncontiguous	Livingston (2); Cayuga (1)	Contiguous (2), Noncontiguous (1)	animal-bovine fecal (2, 2003, SNP range: 30); water-trough (1, 5/2002, SNP range: 33)	30–33
42	6	13.0	1.1	Feb.06	Mar.07	1	Same	Nassau	Contiguous	sink interior (2, 2/2006, 3/2007, SNP range: 41); drain produce area (1, 2/2006, SNP range: 45); shopping cartwheel (1, 2/2006, SNP range: 45); drain raw meat (1, 2/2006, SNP range: 43); floor dry aisle (1, 2/2006, SNP range: 47)	41–47
45	14	174.0	14.5	Oct.04	Apr.19	5	Noncontiguous	Erie (10); Albany (1); Nassau (1); Bronx (1); Kings (1)	Contiguous (1), Noncontiguous (13)	deli meat (1, Kings, 10/2004, SNP: 16–19); environmental swab sponge (1, 10/2017, SNP range: 12–17); dairy case (1, 10/2005, SNP range: 22–23); drain under sink (1, 1/2019, SNP range: 25–26); floor drain (1, 2/2019, SNP range: 32–31); floor near sink (1,2/2019, SNP range: 25–26); slicer (1, 2/2019, SNP range: 32- 31); basket drain (1, 3/2019, SNP range: 29–28); deli slicing room door (1, 10/2017, SNP range: 28–27); deli floor mat (2, 10/2017, SNP range: 24–26); deli cart (1, 10/2017, SNP range: 26–25); floor produce area (1, 9/2005, SNP range: 17–20); basket drain (1, 4/2019, SNP range: 28–29)	12–32
46	31	183.0	15.3	Jul.02	Oct.17	10	Noncontiguous	Ulster (20); Steuben (3); Queens (1); Oneida (1); Cortland (1); Onondaga (1); Kings (1); Suffolk (1); Oswego (1); New York (1)	Same (1), Noncontiguous (30)	deli meat (1; 10/2017; SNP: 33–34); deli meat (1; 5/2016; SNP: 31–32); deli meat (1, Oswego, 11/2002, SNP: 32–33); deli salad (1, Steuben, 9/2004, SNP: 29–30); hot-dogs (1, New York, 7/2002, SNP: 22–23); meat (1; 10/2017; SNP: 30–31); sandwiches (1, Kings, 8/2004, SNP: 30–31); sandwiches (1, Suffolk, 9/2004, SNP: 31–32); shellfish (1, Onondaga, 8/2004, SNP: 20–21); cold storage-dairy walk-in cooler floor (1, 7/2006, SNP: 23–24); dairy case (1, 7/2006, SNP: 30–31); drain-seafood (1,7/2006, SNP: 23–24); drain raw meat (1, 10/2005, SNP:27–28)<br>environmental swab sponge (7; 2/2016; 31–34)<br>environmental swab sponge drain deli (1; 12/2005; SNP: 41–42); environmental swab sponge sink interior (1; 9/2005; SNP: 29–30); sponge (10; 7/2017; 30–34)	20–42
48	11	136.0	11.3	Jul.10	Nov.21	1 ¶	Same (8), Unknown (3)	Richmond (8); Unknown (3)	Noncontiguous (8), Unknown (3)	sponge (1, 11/2021, SNP range: 41–46); Deli sink, 3 basin, interior (2, 7/2010, 10/2007, SNP: 42–47); Deli sink, 1 basin, interior (4, 8/2010, 9/2010, 10/2010, 1/2011, SNP: 41–49); Drain, cold storage room (2, 10/2010, 11/2011, SNP: 45–	41–50

Cluster ID*	No. of non-clinical isolates in NY-cluster	Duration (months)	Duration (years)	Isolation date of the first non-clinical isolate	Isolation date of the last non-clinical isolate	No. of counties	Geolocation connections of non-clinical isolates †	Counties	Geolocation connections of non-clinical isolates to clinical isolates (no. of isolates)	Types of non-clinical isolates (number of samples, county of isolation, isolation month and year, SNP distance ranges to clinical isolates in NY-cluster)	SNP distances to clinical isolates ‡
										50); Floor, cold storage room (1, 12/2010, SNP: 45–50); Trash cans, deli area (1, 1/2011, SNP: 41–46)	
51	1	0.0	0.0	Oct.17	Oct.17	1	NA	Queens	Noncontiguous	sponge swab/deli slicing room door	13–15
52	4	107.0	8.9	Jun.04	May.13	2	Noncontiguous	Kings (3); Ulster (1)	Contiguous (1), Noncontiguous (3)	seafood (1, 5/2013, SNP range: 16); seafood (1, Ulster, 6/2004, SNP: 5); seafood dish (1, 10/2011, SNP range: 20); Scale Tops Slicing, food contact surface (1, 5/2011, SNP range: 26)	5–26
54	2	0.0	0.0	Sep.19	Sep.19	1	Same	Kings	Noncontiguous	deli salad	39–43
56	1	0.0	0.0	Jun.12	Jun.12	1	NA	Queens	Contiguous	cheese	4–5
59	100	220.0	19.1	Jan.00	Feb.19	14 ¶	Noncontiguous (93), Unknown (7)	Bronx (20); Kings (17); Nassau (10); Oneida (8); Queens (8); Erie (6); Monroe (6); New York (4); Broome (4); Suffolk (4); Rockland (3); Onondaga (1); Yates (1); Otsego (1); Unknown (7)	Same (9), Noncontiguous (84), Unknown (7)	cheese (1, Otsego, 2/2004, SNP: 24–25); chicken salad (1, 5/2016, SNP: 39–40); deli meat (1, Queens, 12/2002, SNP: 24–25); deli meat (1, Bronx, 5/2000, SNP: 47–48); deli meat (2, Kings, 8/2000, SNP: 43–45); deli meat (2, 7/2015, 6/2017, SNP: 25–28); Deli Meat (1, 8/2006, SNP: 7–9); deli meat (1, 7/2008, SNP: 28–29); deli meat (1, 6/2015, SNP: 35–36); deli meat (1, 7/2008, SNP: 30–31); deli salad (1, 2008, SNP: 46–47); Deli Salad (1, 8/2006, SNP: 7–10); deli salad (1, 7/2017, SNP: 42–43); deli salad (1, Kings, 9/2000, SNP: 22–23); deli salad (1, Nassau, 10/2003, SNP: 29–30); Engineered seafood (2, Nassau, 7/2002, SNP: 23–24); hot-dogs (1, Bronx, 11/2004, SNP: 16–17); hot-dogs (1, Bronx, 5/2002, SNP: 14–15); sandwiches (1, Queens, 1/2005, SNP: 22–23); sandwiches (1, Suffolk, 1/2004, SNP: 22–23); sandwiches (1, Kings, 12/2003, SNP: 25–26); seafood (1, Queens, 7/2002, SNP: 31–32); seafood (3, Rockland, 1/2000, SNP: 26–33); seafood (1, 7/2013, SNP: 35–36); seafood (1, 10/2018, SNP: 43–46); seafood (1, 9/2014, SNP: 38–39); shellfish spread (1, 1/2013, SNP: 39–40); salad (1, 9/2010, SNP: 24–25); Cutting board, trimming area (1, 6/2004, SNP: 38–39); deli case (3, 11/2005, 6/2006, 3/2007, SNP: 23–48); display case raw meat (1, 10/2005, SNP: 28–29)<br>Door handle (1, 2015, SNP: 32–33); Drain near filet Table (1, 10/2002, SNP: 31–32); drain raw meat (8, 8/2005 (2), 3/2006, 6/2006, 7/2006, 3/2007, 4/2007, 5/2007, SNP: 20–50); drain seafood (2, 6/2006, 3/2007, SNP: 35–49); environment (2, 11/2012, SNP: 29–30); environmental swab sponge (12, 4/2006, 8/2007 (4), 9/2007 (2), 3/2008 (2), 2008 (3), SNP: 23–49); cold storage room shelf (1, 4/2007, SNP: 3031); cutting board (1, 5/2007, SNP: 30–31); drain deli (1, 2/2006, SNP: 40–41); drain produce area (1, 3/2007, SNP: 22–23); floor cold storage room (1, 4/2007, SNP: 20–21); floor dry aisle (2, 6/2006, 3/2007, SNP: 20–25); green cutting board two meat slicer (1, 2/2019, SNP: 45–48); sink interior (4, 3/2007, 4/2007, 5/2007, 12/2007, SNP: 22–46);	1–50

Cluster ID*	No. of non-clinical isolates in NY-cluster	Duration (months)	Duration (years)	Isolation date of the first non-clinical isolate	Isolation date of the last non-clinical isolate	No. of counties	Geolocation connections of non-clinical isolates †	Counties	Geolocation connections of non-clinical isolates to clinical isolates (no. of isolates)	Types of non-clinical isolates (number of samples, county of isolation, isolation month and year, SNP distance ranges to clinical isolates in NY-cluster)	SNP distances to clinical isolates ‡
										surface area of deli equipment wash sink (1, 2/2019, SNP: 44–46); utensil (2, 4/2007, 12/2005, SNP: 28–46); white tubs (1, 11/2001, SNP: 26); floor (1, 3/2001, SNP: 28–29); food contact surface (1, 12/2005, SNP: 44–45); sponge (16, 9/2008 (3), 6/2017 (3), 9/2017 (7), 8/2018 (2), SNP: 1–50)	
60	3	9.0	0.7	Jun.14	Mar.15	1	Same	Otsego	Noncontiguous	red hose in the milk receiving room (1, 6/2014, SNP: 36–39); drain, milk receiving room (2, 12/2014, 3/2015, SNP: 36–39)	36–39
69	1	0.0	0.0	Oct.10	Oct.10	1	NA	Queens	Noncontiguous	deli slicing room door	29–32
70	3	93.0	7.8	Apr.03	Jan.11	1 ¶	NA, Unknown (2)	Suffolk (1), Unknown (2)	Noncontiguous (1), Unknown (2)	sandwiches (1, Suffolk, Jan 2005, SNP: 3–25); Slicer (2, September 2010, Jan 2011 - SNP: 2–25)	2–25
71	1	0.0	0.0	Mar.02	Mar.02	1	NA	Madison	Contiguous	silage/haylage	45–46
73	2	0.0	0.0	Oct.02	Oct.02	1	Same	Westchester	Same	deli meat (1, 10/2002, SNP: 1–23); meat (1, 10/2002, SNP: 0–22)	0–23
74	9	48.0	4.0	Jul.02	Jul.06	8	Noncontiguous	Steuben (2); Cortland (1); Oneida (1); Kings (1); New York (1); Suffolk (1); Onondaga (1); Oswego (1)	Contiguous (1), Noncontiguous (8)	deli meat (1, Oswego, 11/2002, SNP: 47–50); hot-dogs (1, New York, 7/2002, SNP: 37–46); sandwiches (1, Kings, 8/2004, SNP: 31–40); sandwiches (1, Suffolk, 9/2004, SNP: 32–41); shellfish (1, Onondaga, 8/2004, SNP:43–49); cold storage-dairy walk-in cooler floor (1, 7/2006, SNP: 46–49); drain-seafood (1, 7/2006, SNP: 46–49); drain raw meat (1, 10/2005, SNP:50); sink interior (1, 9/2005, SNP: 44–50)	31–50
75	5	16.0	1.3	Mar.09	Jul.10	2	Noncontiguous	Rensselaer (3); Orange (2)	Contiguous (3), Noncontiguous (2)	produce (2, 4/2009, SNP range: 0–6); produce (1, 3/2009, SNP range: 6–9); sprout (2, 7/2010, SNP range: 2–8)	0–9
76	3	9.0	0.8	Jun.14	Mar.15	1	Same	Otsego	Contiguous	red hose in the milk receiving room (1, 6/2014, SNP: 23–30); drain, milk receiving room (2, 12/2014, 3/2015, SNP: 23–30)	23–30
78	75	246.0	20.5	May.01	Nov.21	5 ¶	Contiguous (50), Unknown (25)	Kings (24); Suffolk (14); Bronx (6); Queens (5); New York (1); Unknown (25)	Same (44), Contiguous (6), Unknown (25)	hot-dogs (1, Kings, 6/2007, SNP: 25–43); meat (1, Bronx, 5/2007, SNP: 24–42); seafood (1, Queens, 9/2002, SNP: 19–41); salad (1, 4/2010, SNP: 41–44); floor in deli area (1, 9/2010); Cart (1, 9/2010); Cart wheels (1, 2001); Deli sink, 1 basin, exterior (1, 10/2010); Deli sink, 1 basin, interior (6, 2010, 1011); Counter (1, 12/2010); Drain in microwave room (2, 2002); Drain-produce (1, 3/2007); Drain, cold storage room (4, 2010–2011); Drain, deli area (3, 2010); Floor cleaning equipment (5, 2010–2011); Floor drain in produce prep room (1, 12/2005); Floor in deli area (1, 8/2010); Floor in dry aisle (1, 3/2007); Floor near deli drain (4, 2010); Floor near drain (1, 4/2010); Floor cold storage room (3, 2010–2011); Floor/wall under 3 basin sink (4, 2010); Hoses, floor cleaning (2, 2010–2011); Racks, cold storge room (2, 2010); Scale (1, 8/2010); Standing water (3, 2010); Trash cans, deli area (1, 11/2010); Wall/Floor Juncture (3, 2010); sponge (3, 4/2017(2), 3/2018, SNP: 20–49); floor drain in produce prepared room (1, 12/2005, SNP:	5–50

Cluster ID*	No. of non-clinical isolates in NY-cluster	Duration (months)	Duration (years)	Isolation date of the first non-clinical isolate	Isolation date of the last non-clinical isolate	No. of counties	Geolocation connections of non-clinical isolates †	Counties	Geolocation connections of non-clinical isolates to clinical isolates (no. of isolates)	Types of non-clinical isolates (number of samples, county of isolation, isolation month and year, SNP distance ranges to clinical isolates in NY-cluster)	SNP distances to clinical isolates ‡
										36–39); sink interior (1, 2/2006, SNP: 22–39); drain produce area (2, 3/2006 and 3/2007, SNP: 38–43); drain deli (1, 3/2007, SNP: 37–40) food dry aisle (1, 3/2007, 40–43); environmental swab sponge (7, 10/2007, 2008, 10/2021 (5), 11/2021 (4), SNP: 5–49)	
79	8	3.0	0.3	Feb.18	May.18	1	Same	Rensselaer	Contiguous	raw milk (1, 2/2018, SNP: 26–30); green scrubby (1, 5/2018, SNP: 26–30); environmental swab sponge jetter cup (1, 5/2018, SNP: 24–28); new plastic bucket interior (1, 5/2018, SNP:24–28); environmental swab sponge parlor drain (1, 5/2018, SNP: 24–28); bucket hook jetter cup shield (1, 5/2018, SNP: 26–30); holding area standing water (1, 5/2018, SNP: 32–36)	24–36
81	99	229.0	19.1	Jan.00	Feb.19	14 ¶	Noncontiguous (96), Unknown (3)	Bronx (20); Kings (18); Nassau (10); Oneida (9); Queens (8); Erie (6); Monroe (6); Suffolk (4); New York (4); Broome (4); Rockland (3); Onondaga (1); Yates (1); Otsego (1); Unknown (3)	Same (27), Noncontiguous (69), Unknown (3)	cheese (1, Otsego, 2/2004, SNP: 31–39); chicken salad (1, 5/2016, SNP: 33–34); deli meat (1, Queens, 12/2002, SNP: 4–37); deli meat (1, Bronx, 5/2000, SNP: 27–42); deli meat (2, Kings, 8/2000, SNP: 23–39); deli meat (1, 8/2006, SNP: 25–45); deli meat (1, 7/2008, SNP: 22–41); deli meat (1, 7/2008, SNP: 24–43); deli meat (2, 7/2015(1), 6/2017(1), SNP: 4–40); deli meat (1, 6/2015, SNP: 29–38); deli salad (1, Kings, 9/2000, SNP: 16–35); deli salad (1, 7/2017, SNP: 46–47); deli salad (1, Nassau, 10/2003, SNP: 23–42); deli salad (1, ?/2008, SNP: 2–41); deli salad (1, 8/2006, SNP: 25–45); Engineered seafood (2, Nassau, 7/2002, SNP: 17–36); hot-dogs (1, Bronx, 11/2004, SNP: 20–39); hot-dogs (1, Bronx, 5/2002, SNP: 18–37); sandwiches (1, Queens, 1/2005, SNP: 28–47); sandwiches (1, Suffolk, 1/2004, SNP: 28–47); sandwiches (1, Kings, 12/2003, SNP: 31–50); seafood (1, Queens, 7/2002, SNP: 25–40); seafood (3, Rockland, 1/2000, SNP: 21–44); seafood (1, 7/2013, SNP: 29–48); seafood (2, 10/2009 (1), 9/2014(1), SNP: 32–50); seafood (1, 10/2018, SNP: 37–40); salad (1, 9/2010, SNP: 28–47); spread (1, 1/2013, SNP: 33–34); drain raw meat (5, 8/2005 (2), 6/2006, 7/2006 (1), 5/2007 (1), SNP: 18–44); food contact surface (1, 12/2005, SNP: 0–39); sponge (16, 10/2008 (3), 6/2017 (3), 9/2017 (8), 8/2018 (2), SNP: 2–46); sink interior (4, 12/2005 (1), 3/2007 (1), 4/2007 (1), 5/2007 (1), SNP: 1–46); utensil (2, 12/2005 (1), 4/2007 (1), SNP: 1–50); drain deli (1, 2/2006, SNP: 34–35); floor dry aisle (2, 6/2006, 3/2007 (1), SNP: 14–37); drain seafood (2, 6/2006, 3/2007, SNP: 1–44); Drain near fillet table (1, 2002); White tubs - clean (Nova tubs) (1,11/ 2001); floor (1, 3/2001); deli case (3, 11/2005, 6/2006, 3/2007, SNP: 1–42); display case raw meat (1, 10/2005, SNP: 22–37); environment (4, 2001 (2), 2002 (1), SNP: 20–41); environmental swab sponge	0–50

Cluster ID*	No. of non-clinical isolates in NY-cluster	Duration (months)	Duration (years)	Isolation date of the first non-clinical isolate	Isolation date of the last non-clinical isolate	No. of counties	Geolocation connections of non-clinical isolates †	Counties	Geolocation connections of non-clinical isolates to clinical isolates (no. of isolates)	Types of non-clinical isolates (number of samples, county of isolation, isolation month and year, SNP distance ranges to clinical isolates in NY-cluster)	SNP distances to clinical isolates ‡
										(13, 3/2006, 4/2006, 8/2007 (4), 9/2007 (2), 3/2008 (2), 2008 (3), SNP: 7–50); cold storage room shelf (1, 4/2007, SNP: 34–35); cutting board (1, 5/2007, SNP: 20–43); drain produce area (1, 3/2007, SNP: 16–35); floor cold storage room (1, 4/2007, SNP: 24–43); green cutting board two meat slicer (1, 2/2019, SNP: 25–42); surface area of deli equipment wash sink (2, 2/2019, 24–40); Cutting board, trimming area (5, 2004)	
82	5	145.0	12.1	Nov.02	Dec.14	4	Noncontiguous	Queens (2); Columbia (1); Otsego (1); Albany (1)	Contiguous (4), Noncontiguous (1)	hot-dogs (1, queens, 7/2007, SNP: 14–25); hot-dogs (1, queens, 8/2007, SNP: 14–23); swab, main drain in blue processing room (1, 12/2013, SNP: 17–26); drain, processing room (1, 12/2014, SNP: 10–23); water-urban-flowing water (1, 11/2002, SNP: 9–20)	8–26
83	3	43.0	3.6	Aug.03	Mar.07	3	Noncontiguous	Suffolk; Bronx; Queens	Same (2), Contiguous (1)	deli salad (1, Bronx, 9/2004, SNP: 2–14); deli salad (1, Queens, 8/2003, SNP: 15–21); environmental swab sponge floor cold storage room (1, Suffolk, March 2007, SNP: 13–20)	2–21
84	5	69.0	5.8	Sep.02	Jun.08	2	Contiguous	Nassau (2); New York (2); Queens (1)	Same (3), Contiguous (2)	deli meat (2, 9/2002, New York, SNP: 0–7); deli meat (1, 6/2008, SNP: 3–7); environmental swab sponge (2, 2008, SNP: 7–15)	0–15
85	5	195.0	16.3	Jun.02	Sep.18	4 ¶	Noncontiguous (4), Unknown (1)	Suffolk (1); Tompkins (1); Tioga (1); Steuben (1); Unknown (1)	Same (1), Contiguous (3), Unknown (1)	grass sample by standing water in pasture (1,6/2003, SNP: 9–25); drain-raw meat (1, 7/2006, SNP: 5–20); soil (2, 9/2018, 2013, SNP: 9–31); animal bovine (1, 6/2002, SNP: 11–27)	5–31

*Cluster IDs 1 to 85 were assigned to NY-clusters identified in this study.
†NA, not applicable, was used to specify NY-clusters including one non-clinical isolate only. The number of non-clinical isolates with no exact county-level geolocation was given in parenthesis.
‡Single Nucleotide Polymorphism (SNP) distance here indicates the SNP distances between clinical isolates in NY-clusters and non-clinical isolates.
§The isolation month of either first or last non-clinical isolate is not known.
¶The exact county-level geolocation is missing for at least one non-clinical isolate in the NY-cluster.

Appendix Table 6. Non-clinical isolates identified in NY-clusters in New York State between 2000 and 2021 (non-clinical isolates differing from NY-clinical isolates by ≤50 SNP differences)

Cluster ID*	No. of non-clinical isolates	Isolation date of the first isolate	Isolation date of the last isolate	No. of counties	Geolocation connections of non-clinical isolates †	Geolocation connections of non-clinical isolates to clinical isolates (no. of isolates)	Types of isolates	SNP distance range‡	Relatedness (H, C, L) §
Same county (n = 4)									
Small clusters (n = 3)									
2	3	Jan 2004	Oct 2012	2	Noncontiguous	Noncontiguous (3)	2 food, 1 environmental	41–47	L
3	25	Aug 2003	July 2017	5	Noncontiguous	Same (20), Noncontiguous (5)	5 food, 20 environmental	0–49	H (20), L (5)
4	4	Jun 2002	2013 ¶	4	Noncontiguous	Noncontiguous (4)	1 animal, 3 environmental	26–38	L
Medium clusters (n = 1)									
18	14	Oct 2004	Feb 2019	5	Noncontiguous	Same (10) Noncontiguous (4)	1 food, 13 environmental	1–31	H (10), L (4)
Contiguous counties (n = 7)									
Small clusters (n = 5)									
21	1	Jul 2003	Jul 2003	1	NA	Same (1)	1 food	11–12	C
23	5	Aug 2006	May 2021	2 #	Noncontiguous, unknown (1)	Contiguous (2), Noncontiguous (2), unknown (1)	1 animal, 1 food, 3 environmental	29–42	L
26	5	Aug 2006	May 2021	2 #	Noncontiguous, unknown (1)	Same (2), Noncontiguous (2), unknown (1)	1 animal, 1 food, 3 environmental	30–42	L
27	10	Jul 2002	Jul 2006	8	Noncontiguous	Noncontiguous (10)	5 food, 5 environmental	33–50	L
29	3	Apr 2012	Nov 2021	1	Same	Noncontiguous (3)	2 food, 1 environmental	2–10	H
Medium clusters (n = 2)									
32	3	Apr 2003	Jan 2009	3	Noncontiguous	Same (1), Noncontiguous (2)	3 food	2–19	H
33	2	Jan 2019	Jan 2019	1	Same	Same (2)	2 environmental	4–8	H
Noncontiguous counties (n = 26)									
Small clusters (n = 15)									
35	2	2008 ¶	Sep 2008	2	Noncontiguous	Noncontiguous (2)	2 environmental	26–29	L
36	3	May 2002	2003 ¶	2	Noncontiguous	Contiguous (2), Noncontiguous (1)	2 animal, 1 environmental	30–33	L
42	6	Feb 2006	Mar 2007	1	Same	Contiguous (6)	6 environmental	41–47	L
45	14	Oct 2004	Apr 2019	5	Noncontiguous	Contiguous (1), Noncontiguous (13)	1 food, 13 environmental	12–32	C (3), L (11)
46	31	Jul 2002	Oct 2017	10	Noncontiguous	Same (1), Noncontiguous (30)	9 food, 22 environmental	20–42	L
48	11	Jul 2010	Nov 2021	1 #	Same, unknown (3)	Noncontiguous (8), unknown (3)	11 environmental	41–50	L
51	1	Oct 2017	Oct 2017	1	NA	Noncontiguous (1)	1 environmental	13–15	C
52	4	Jun 2004	May 2013	2	Noncontiguous	Contiguous (1), Noncontiguous (3)	3 food, 1 environmental	5–26	H (1), C (2), L (1)
54	2	Sep 2019	Sep 2019	1	Same	Noncontiguous (2)	1 food	39–43	L
56	1	Jun 2012	Jun 2012	1	NA	Contiguous (1)	1 food	4–5	H

Cluster ID*	No. of non-clinical isolates	Isolation date of the first isolate	Isolation date of the last isolate	No. of counties	Geolocation connections of non-clinical isolates †	Geolocation connections of non-clinical isolates to clinical isolates (no. of isolates)	Types of isolates	SNP distance range‡	Relatedness (H, C, L) §
59	100	Jan 2000	Feb 2019	14 #	Noncontiguous, unknown (7)	Same (9), Noncontiguous (84), unknown (7)	31 food, 69 environmental	1–50	H (3), C (2), L (95)
60	3	Jun 2014	Mar 2015	1	Same	Noncontiguous (3)	3 environmental	36–39	L
69	1	Oct 2010	Oct 2010	1	NA	Noncontiguous (1)	1 environmental	29–32	L
70	3	Apr 2003	Jan 2011	1 #	NA, unknown (2)	Noncontiguous (1), unknown (2)	1 food, 2 environmental	2–25	H
71	1	Mar 2002	Mar 2002	1	NA	Contiguous (1)	1 environmental	45–46	L
Medium clusters (n = 7)									
73	2	Oct 2002	Oct 2002	1	Same	Same (2)	2 food	0–23	H
74	9	Jul 2002	Jul 2006	8	Noncontiguous	Contiguous (1), Noncontiguous (8)	5 food, 4 environmental	31–50	L
75	5	Mar 2009	Jul 2010	2	Noncontiguous	Contiguous (3), Noncontiguous (2)	5 food	0–9	H
76	3	Jun 2014	Mar 2015	1	Same	Contiguous (3)	3 environmental	23–30	L
78	75	May 2001	Nov 2021	5 #	Contiguous, unknown (25)	Same (44), Contiguous (6), unknown (25)	4 food, 71 environmental	5–50	L
79	8	Feb 2018	May 2018	1	Same	Contiguous (8)	1 food, 7 environmental	24–36	L
81	99	Jan 2000	Feb 2019	14 #	Noncontiguous, unknown (3)	Same (27), Noncontiguous (69), unknown (3)	34 food, 65 environmental	0–50	H (13), C (3), L (83)
Large clusters (n = 4)									
82	5	Nov 2002	Dec 2014	4	Noncontiguous	Contiguous (4), Noncontiguous (1)	2 food, 3 environmental	8–26	H (1), L (4)
83	3	Aug 2003	Mar 2007	3	Noncontiguous	Same (2), Contiguous (1)	2 food, 1 environmental	2–21	H (1), C (2)
84	5	Sep 2002	Jun 2008	2	Contiguous	Same (3), Contiguous (2)	3 food, 2 environmental	0–15	H
85	5	Jun 2002	Sep 2018	4 #	Noncontiguous, unknown (1)	Same (1), Contiguous (3), unknown (1)	1 animal, 4 environmental	5–31	H (4), C (1)

* Cluster IDs 1 to 85 were assigned to NY-clusters identified in this study.
† NA, not applicable, was used to specify NY-clusters including one non-clinical isolate only. The number of non-clinical isolates with no exact county-level geolocation was given in parenthesis.
‡ Single Nucleotide Polymorphism (SNP) distance here indicates the SNP distances between clinical isolates in NY-clusters and non-clinical isolates.
§ "Highly-related (H)" means that the number of SNP differences between non-clinical isolates to the most related clinical isolates in a NY-cluster is ≤10; "Closely-related (C)" means that the number of SNP differences between non-clinical isolates to the most related clinical isolates in a NY-cluster is >10 but ≤20; "Loosely-related (L)" means that the number of SNP differences between non-clinical isolates to the most related clinical isolates in a NY-cluster is >21 but ≤50.
¶ The isolation month of either first or last non-clinical isolate is not known.
# The exact county-level geolocation is missing for at least one non-clinical isolate in the NY-cluster.

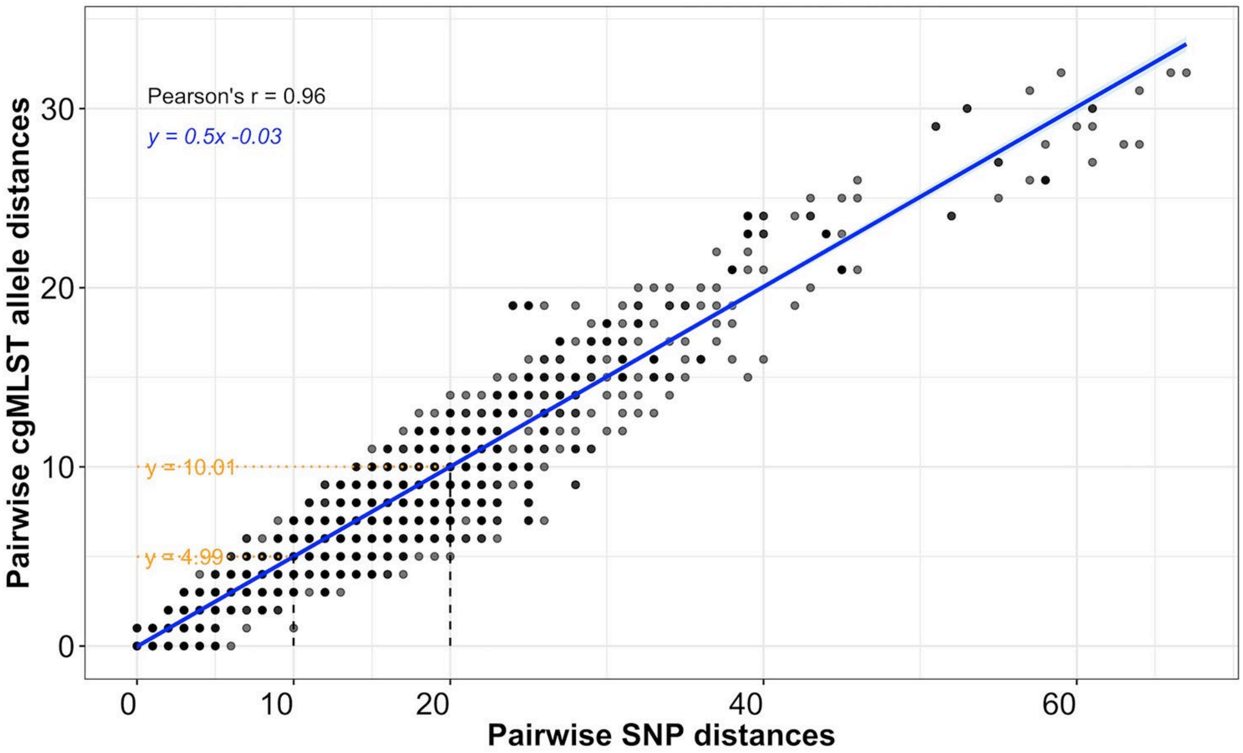

Appendix Figure 1. Correlation between pairwise SNP distances and cgMLST allele distances among Lm isolate pairs in NY-clusters. Each point represents a unique isolate pair. A strong positive correlation was observed between SNP and allele distances (Pearson's r = 0.96). The blue line represents the linear regression fit. Dashed vertical lines are drawn at SNP thresholds to define highly related isolates (SNP ≤10) and SNP threshold used for clustering in this study (SNP ≤20). Corresponding horizontal dotted lines project these thresholds on the regression line to show the expected allele distances at those SNP values.

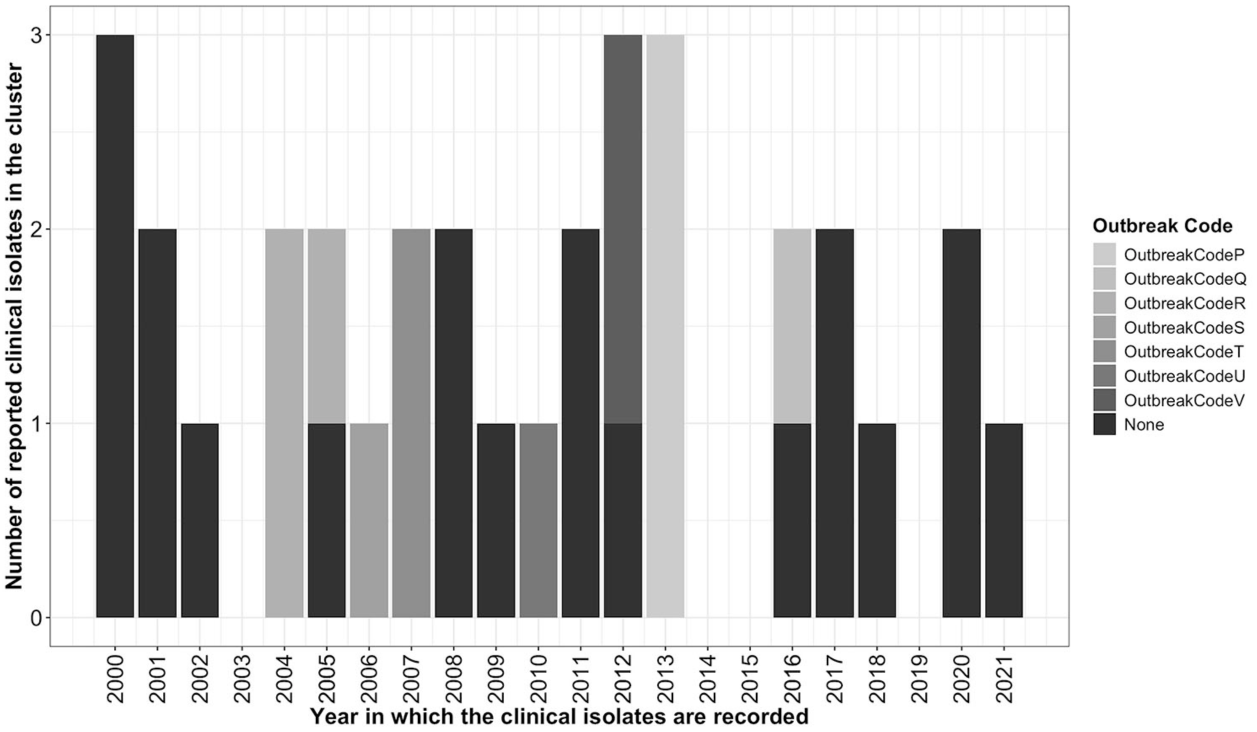

Appendix Figure 2. Epidemic curve for the highly persistent NY-cluster 85 on a yearly basis from 2000 to 2021. Black represents cases that were not assigned an outbreak code (shown as none), whereas the grey shades represent cases that were assigned 7 different outbreak codes (Outbreak Codes P to V).
